# Research Progress on the Association Between Sarcopenic Obesity and Atherosclerosis: Current Status and Challenges

**DOI:** 10.3390/jcm14228148

**Published:** 2025-11-17

**Authors:** Kai Yang, Wei Yang, Si-Cong Si, Jia Liu, Yi-Xin Ma, Huan Zhao

**Affiliations:** Department of Geriatrics, Xuanwu Hospital, Capital Medical University, National Clinical Research Center for Geriatric Diseases, Beijing 100053, China; 24kyang@mail.ccmu.edu.cn (K.Y.); sisicong2025@outlook.com (S.-C.S.); snail_lj@hotmail.com (J.L.); mayixin@xwhosp.org (Y.-X.M.); zhaohuanys@outlook.com (H.Z.)

**Keywords:** sarcopenic obesity, atherosclerosis, diagnostic criteria, association, pathophysiology

## Abstract

Sarcopenic obesity (SO) is a syndrome characterized by a gradual reduction in skeletal muscle mass, strength, and function coupled with excessive fat accumulation, which considerably increases the risk of metabolic disorders and atherosclerotic cardiovascular disease. Owing to its extensive influence on the health of elderly individuals and distinct pathophysiological mechanisms, SO should be considered an independent clinical condition. Atherosclerosis, the fundamental pathophysiological underpinning of atherosclerotic cardiovascular disease, has garnered increased interest because of its association with SO. Existing research indicates that SO may synergistically promote atherosclerosis development through multiple pathways, including hormonal dysregulation, adipo-myokine imbalance, insulin resistance, chronic low-grade inflammation, and lipid metabolic abnormalities. The current literature gaps predominantly encompass the absence of standardized diagnostic criteria for SO, inconsistent results in studies investigating the relationship between SO and atherosclerosis, and inadequate causal validation. Studies indicate associations between SO and carotid atherosclerosis, coronary atherosclerosis, arterial stiffness, and 10-year atherosclerotic cardiovascular disease risk; however, conclusions remain inconsistent, and most studies are cross-sectional. Additionally, this field has insufficient focus on peripheral atherosclerosis, such as in the lower extremities. Moreover, the pathophysiological mechanisms remain unclear. A complex vicious cycle potentially exists among decreased muscle mass and function, fat accumulation, and atherosclerosis, a relationship that has not received sufficient attention. Therefore, this review aims to integrate existing evidence, summarize advances in diagnostic criteria for SO, review the epidemiological association between sarcopenic obesity and atherosclerosis, and analyze the reasons for heterogeneity in conclusions. It further explores potential pathophysiological mechanisms, delving into the vicious cycle among declining muscle mass and function, fat accumulation, and atherosclerosis. Finally, this review proposes future research directions, including diagnostic standardization, in-depth mechanism exploration, conducting prospective cohort studies to validate causal relationships, and developing intervention targets for SO–Atherosclerosis comorbidity.

## 1. Introduction

The European Society for Clinical Nutrition and Metabolism (ESPEN) and the European Association for the Study of Obesity (EASO) have defined sarcopenic obesity (SO) as the co-existence of excess adiposity and low muscle mass/function. Based on etiology, it can be classified into primary and secondary sarcopenic obesity [1]. Risk factors for SO encompass multiple aspects: metabolic changes resulting from sedentary lifestyles, adipose tissue dysfunction, comorbidities (acute and chronic diseases), and the aging process [2]. The organizations emphasize that SO is not a simple combination of sarcopenia and obesity. Sarcopenia is defined by reduced skeletal muscle mass (age-related limb skeletal muscle mass) and function, but muscle changes must be considered within the context of obesity [1]. Specifically, SO focuses more on the decline in relative skeletal muscle mass (standardized by body weight). Furthermore, SO is not limited to older individuals; it can also occur in younger obese patients experiencing acute or chronic diseases or rapid weight fluctuations [1]. Moreover, the bidirectional pathogenic interaction between skeletal muscle mass/function loss and body fat accumulation triggers multiple complications—including frailty, disability, metabolic disorders, cardiovascular disease (CVD), and cancer—significantly elevating mortality risk. This has emerged as a critical public health challenge [3,4]. The prevalence of SO is significantly affected by diagnostic criteria [5]. Research suggests that the worldwide prevalence of SO in older individuals is approximately 11%, with this rate consistently rising each year in conjunction with population aging [6,7].

Atherosclerosis is a chronic inflammatory condition characterized by endothelial dysfunction, lipid deposition, smooth muscle cell proliferation, fibrous matrix hyperplasia, and infiltration of inflammatory cells, culminating in plaque formation [8]. In the context of global aging, atherosclerosis acts as a catalyst for the progression of atherosclerotic cardiovascular disease (ASCVD), such as acute coronary syndrome, diverse forms of angina pectoris, ischemic stroke, transient ischemic episodes, and peripheral artery disease. The overall incidence of ASCVD events continues to increase [9,10]. The elevated prevalence, disability rate, and mortality linked to atherosclerosis and its consequent ASCVD place a significant burden on patients, their families, and society. Nonetheless, early warning indicators for atherosclerosis are still in the exploratory stage, hence complicating prevention and management initiatives [11,12]. Consequently, the prompt identification and intervention of atherosclerosis risk factors, along with efficacious preventative and therapeutic strategies, are essential for diminishing the prevalence of cardiovascular disease and enhancing patients’ quality of life.

The relationship between SO and atherosclerosis has recently attracted interest. Nonetheless, because of research limitations—such as uneven diagnostic criteria, heterogeneous study populations, comorbidities, and sex disparities—the association between SO and atherosclerosis remains contentious. The causal relationship remains unestablished, and the underlying pathophysiological mechanisms of this correlation are uncertain. While the current understanding of obesity and atherosclerosis may partially elucidate the relationship between SO and atherosclerosis, SO cannot be seen as a straightforward aggregation of sarcopenia and obesity. The “obesity paradox” has led academics to discuss the importance of muscle-fat interactions. Recent studies have indicated that SO may facilitate the initiation and progression of atherosclerosis via various mechanisms, including hormonal abnormalities, insulin resistance (IR), chronic low-grade inflammation, and dyslipidemia [13,14,15,16,17]. A complex vicious cycle may exist among muscle loss, fat accumulation, and atherosclerosis—a link not yet thoroughly investigated in prior research. Clarifying the relationship and mechanisms between SO and atherosclerosis is crucial for the early prevention and treatment of ASCVD. This review intends to encapsulate recent developments in SO diagnostic criteria, consolidate research on their correlations, and investigate probable mechanisms—specifically, the detrimental cycle involving muscle atrophy, adipose tissue deposition, and atherosclerosis. This review establishes a theoretical framework for enhancing future research and delineates prospective directions for investigation.

## 2. Literature Search Strategy

A systematic search of PubMed, Web of Science, and Scopus databases was conducted to review current diagnostic criteria for SO, research progress on the association between SO and atherosclerosis, and its pathophysiological mechanisms. Search terms included sarcopenic obesity/SO, diagnostic criteria/diagnosis, atherosclerosis/carotid atherosclerosis/coronary artery atherosclerosis/arterial stiffness/atherosclerotic cardiovascular disease, association, pathophysiology/mechanism, and their combinations. The search covered the period from database inception to September 2025, with irrelevant literature excluded.

## 3. Obesity Paradox Highlights the Importance of Comprehensively Evaluating Body Composition and Physical Function

With advancing age, significant changes occur in body composition, primarily manifested as a relative increase in adipose tissue and a gradual decrease in muscle mass, while total body weight and body mass index (BMI) may remain relatively stable [18,19]. Although obesity is widely recognized as a major risk factor for the onset and mortality of cardiovascular disease in adults, its impact on the elderly remains controversial. Research indicates that overweight status (BMI ≥ 25 kg/m^2^) may be associated with improved survival rates among cardiovascular disease patients—a phenomenon termed the obesity paradox [20,21].

The obesity paradox describes the observation that overweight or obese individuals often exhibit higher survival rates than normal-weight or underweight patients in certain chronic diseases, such as cardiovascular disease and chronic kidney disease. This phenomenon is particularly pronounced in the field of cardiovascular disease [22,23]. For example, Angeras et al. (2013) found in the Swedish Coronary Angiography and Angioplasty Registry that, based on BMI grouping, overweight and obese patients had higher survival rates in acute coronary syndrome compared to normal-weight and underweight patients [20]. Similarly, Lavie et al. (2012) observed in a study of patients with stable coronary artery disease that mortality was highest in those with low lean mass index (LMI)/low body fat (BF) and lowest in those with high LMI/high BF [21].

The mechanism underlying the obesity paradox remains unclear. Research suggests that obesity may increase metabolic reserves, enabling better adaptation to metabolic stress associated with chronic diseases [23]. Adipose tissue secretes protective adipokines [24,25], thereby improving cardiovascular disease outcomes. Additionally, most scholars believe the inherent limitations of BMI as a single indicator are a key factor [26]. BMI fails to reflect fat distribution and cannot accurately distinguish between adipose and muscle tissue, despite these two components potentially exerting opposite effects on disease and mortality risks [27,28]. Individuals with normal BMI may harbor elevated metabolic risks due to excessive visceral fat, while those with high muscle mass may be misclassified as obese based on elevated BMI [29]. Studies indicate that at equivalent BMI levels, individuals with low muscle mass exhibit higher body fat percentages, higher diabetes prevalence, and increased mortality risk, whereas those with high muscle mass demonstrate significantly reduced mortality risk. This suggests muscle mass modulates the relationship between BMI and mortality, inversely correlating with mortality risk [30].

Furthermore, beyond BMI, McAule et al. (2012) demonstrated that this paradox also exists among patients with higher waist circumference and greater body fat percentage [31]. They showed that coronary heart disease patients with larger waist circumferences or higher body fat percentages but normal cardiopulmonary function did not exhibit increased risks of all-cause mortality or cardiovascular disease mortality [31]. This suggests that assessing mortality risk based solely on obesity may be misleading; physical fitness and function should be considered concurrently [31,32].

During the aging process, even with stable total body weight, body composition may undergo significant changes: fat mass (particularly visceral fat) increases, while skeletal muscle mass progressively decreases [18,19]. Visceral obesity or central obesity exerts a more pronounced impact on metabolic health than total body weight alone [33]. When obesity coexists with muscle loss, the mutual negative effects of sarcopenia and obesity may diminish obesity’s metabolic buffering capacity. For instance, Shi et al. (2024) found that low appendicular skeletal muscle mass index (ASMI, ASM/height^2^), low grip strength, or low appendicular skeletal muscle mass to visceral fat area ratio (ASM/VFA) significantly increased the 10-year risk of ASCVD in women aged 65 years and older [34].

The obesity paradox does not negate the harms of obesity, but rather cautions us that when assessing cardiovascular risk, we must go beyond single obesity indicators like BMI. Instead, we must comprehensively evaluate body composition (such as fat distribution, visceral fat, and muscle mass) as well as physical function. This approach may help us better understand the relationship between obesity and cardiovascular disease prognosis [24]. The significance of SO research lies precisely here: it underscores that the qualitative and functional interactions between muscle and fat are more critical than mere weight or obesity status. Recognizing SO as a distinct clinical syndrome facilitates the identification of high-risk individuals masked by normal BMIs, enabling more precise risk assessment and early intervention for atherosclerotic cardiovascular disease.

## 4. Independence and Health Implications of Sarcopenic Obesity

Notably, not all obese elderly individuals develop sarcopenia, as weight-bearing exercise frequently activates muscle mechanoreceptors, facilitating the production of growth factors and muscle growth [35]. However, in patients with SO, this adaptive physiological mechanism may be compromised, resulting in excessive fat storage without concomitant enhancements in muscle growth and function [36]. Consequently, an imbalance between muscle and fat may arise during various metabolic conditions or fluctuations in weight, potentially instigating SO. For example, weight reduction is frequently advised for obese patients with type 2 diabetes mellitus; nevertheless, this approach may impair muscle strength and function, thus facilitating SO and consequently increasing health hazards [37,38,39]. This is due to the established correlation between SO and all-cause mortality in the elderly [40,41]. Recent prospective cohort data demonstrated that SO is a substantial predictor of all-cause mortality in hospitalized older patients (HR = 2.84), exhibiting a greater risk than sarcopenia (HR = 1.93) and remaining independent of age, sex, and BMI [42].

SO is a more serious threat to aged individuals than sarcopenia or obesity alone. Research has demonstrated that, compared with sarcopenia or obesity alone, SO is associated with an increased risk of negative outcomes in elderly individuals, including falls, disability, fractures, dementia, cancer, metabolic disorders, and cardiovascular disease [43,44,45,46,47,48]. In the cardiovascular area, SO considerably increases the risk of cardiovascular risk factors—such as insulin resistance, metabolic syndrome, and abnormalities in glucose and lipid metabolism—more than obesity or sarcopenia alone [41,48]. A meta-analysis of 106 clinical studies revealed that SO correlates with a heightened incidence of stroke, myocardial infarction, angina pectoris, and heart failure [49]. Research applying China’s CHARLS database indicated that individuals with SO exhibit a 1.47-fold increased risk of cardiovascular disease and a 1.48-fold heightened risk of heart disease in comparison to those with sarcopenia alone [45]. However, a minority of studies do not support a more pronounced cardiovascular risk from SO. As a result, the synergistic relationship between muscle and fat has garnered increased interest. The accumulation of body fat results in a vicious cycle with declining skeletal muscle mass and function through the cross-talk of myokines and adipokines, inflammatory responses, and metabolic pathways, exacerbating adverse clinical outcomes [50,51].

International consensus clearly indicates that SO is not merely a combination of sarcopenia and obesity but also an independent clinical illness characterized by distinct pathological characteristics, including an imbalanced muscle-to-fat ratio and metabolic abnormalities [52,53]. Clarifying this concept enables targeted interventions, including increasing muscle mass, increasing muscle strength, decreasing body fat, improving physical function, reducing illness risk, and enhancing patient prognosis.

## 5. Diagnosis of Sarcopenic Obesity

The absence of standardized diagnostic criteria for SO poses significant challenges in clinical practice. Consequently, multiple international organizations have developed diagnostic consortia, each proposing tailored screening and diagnostic methods while emphasizing the need for ethnicity-specific cutoffs adjusted for age and sex (Table 1).

### 5.1. ESPEN/EASO Consensus

The diagnostic process jointly proposed by the European Society for Clinical Nutrition and Metabolism (ESPEN) and the European Association for the Study of Obesity (EASO) includes three stages: screening, diagnosis, and staging [3]. ESPEN/EASO is the first way to distinguish between primary and secondary SO, which helps in understanding the pathogenesis of SO and developing personalized intervention strategies. After the diagnosis of SO is confirmed, the condition is classified into stage I (without complications) and stage II (with at least one complication, such as metabolic diseases or cardiovascular diseases) on the basis of the presence of comorbidities, which facilitates the assessment of disease severity [3]. With respect to muscle mass measurement, ESPEN/EASO recommends the use of appendicular lean mass adjusted for body weight (ALM/W) and total skeletal muscle mass adjusted for body weight (SMM/W) [3]. This is because, when total body weight and fat mass are greater without a reduction in absolute muscle mass, the relative decrease in muscle mass may have significant clinical and functional consequences, and the above indices effectively capture this characteristic [54].

### 5.2. JWGSO Consensus

The Japanese Working Group on SO (JWGSO) has innovatively introduced the Finger Ring test into sarcopenia screening. This test involves encircling the thickest part of the nondominant calf with the index finger and thumb, and a positive result is indicated when the calf circumference is equal to or smaller than the circle formed by the fingers [55]. The Finger Ring test enhances the convenience of the screening process. With respect to muscle mass measurement, this consensus recommends the use of limb skeletal muscle mass adjusted for BMI (LSMM/BMI), a measure that better aligns with the body composition characteristics of the elderly population in Japan and has greater applicability [56].

**Table 1:** Recommended screening indicators and diagnostic criteria for sarcopenic obesity by various organizations.

Organizations [Reference]	Proposed Consensus Time	Screening Indicators		Diagnostic Criteria	
		Sarcopenia	Obesity	Sarcopenia	Obesity
ESPEN/EASO [1]	2022	Clinical symptoms or Clinical suspicion or SARC-F > 4	BMI (≥30 kg/m^2^ for CP, ≥27.5 kg/m^2^ for AP) or WC (M ≥ 102 cm for CP, F ≥ 88 cm for CP; M ≥ 78 cm for AP; F ≥ 72 cm for AP)	Muscle strength: Hand Grip (M < 27 kg for CP; F < 16 kg for CP; M < 28 kg for AP; F < 18 kg for AP); Physical performance: 5-Times Sit-to Stand Test ≥ 17 s; Muscle mass measurement: ALM/W by DXA (M < 25.7%; F < 19.4%) or SMM/W by BIA (M < 37.0%, F < 27.6%)	BFP(M > 31% for CP, F > 43% for CP; M > 29% for AP, F > 41% for AP)
JWGSO [56]	2024	Calf circumference (M < 34 cm, F < 33 cm) or SARC-F > 4, SARC-CalF > 11 or Finger ring test or Clinical symptoms or Clinical suspicion	WC (M > 85 cm, F > 90 cm) or BMI > 25 kg/m^2^	Muscle strength: Hand Grip (M < 28 kg, F < 18 kg); Physical performance: 5-Times Sit-to Stand Test > 12 s; Muscle mass measurement: ASM/BMI (M < 0.789 kg/BMI; F < 0.512 kg/BMI) by DXA or BIA	VFA > 100 cm^2^ or BFP (M ≥ 20%, F ≥ 30%)
AOASO/IAGG-AOR [52]	2025	Calf circumference (M < 34 cm, F < 33 cm) or SARC-F > 4, SARC-CalF > 11 or Finger Ring test or Clinical suspicion	BMI * OR WC *	Muscle strength: Hand Grip (M < 28 kg, F < 18 kg); Physical performance: 6-Meter Walk < 1.0 m/s or 5-Times Sit-to Stand Test > 12 s or SPPB ≤ 9; Muscle mass measurement: ASMI by DXA (M < 7.0 kg/m^2^, F < 5.4 kg/m^2^) or BIA (M < 7.0 kg/m^2^, F < 5.7 kg/m^2^)	VFA * by DXA/CT/MRI or BFP * by BIA/DXA If the above measurements are not feasible, consider Waist Circumference, Waist/Hip ratio or BMI

Abbreviations: ALM/W, Appendicular Lean Mass/Weight; AOASO, Asia-Oceania Association for the Study of Obesity; AP, Asian Populations; ASMI, Appendicular Skeletal Muscle Mass Index; BFP, Body Fat Percentage; BIA, Bioelectrical Impedance Analysis; BMI, Body Mass Index; CP, Caucasian Populations; CT, Computed Tomography; DXA, Dual-energy X-ray Absorptiometry; EASO, European Association for the Study of Obesity; ESPEN, European Society for Clinical Nutrition and Metabolism; F, Female; IAGG-AOR, International Association of Gerontology and Geriatrics Asia-Oceania Region; JWGSO, Japanese Working Group on Sarcopenic Obesity; M, Male; MRI, Magnetic Resonance Imaging; SARC-CalF, SARC-F combined with Calf Circumference; SARC-F, Strength, Assistance with walking, Rising from a chair, Climbing stairs, Falls; SMM/W, Skeletal Muscle Mass/Weight; SPPB, Short Physical Performance Battery; VFA, Visceral Fat Area; WC, Waist Circumference; *, Adapted to the cut-offs of each country or population.

### 5.3. AOASO/IAGG-AOR Consensus

The consensus from the Asia-Oceania Association for the Study of Obesity (AOASO) and the International Association of Gerontology and Geriatrics Asia/Oceania Region (IAGG-AOR) emphasizes the key role of central obesity (abdominal obesity) in the diagnosis of SO [52]. Research has shown that central obesity (such as increased waist circumference and waist-to-hip ratio) is a better predictor of cardiovascular and metabolic risk than BMI is [57,58,59]. Additionally, indicators such as the visceral fat area (VFA) and body fat percentage (BFP) provide greater accuracy in evaluating visceral fat than traditional anthropometric measurements do [60,61]. Given the unique body fat distribution and genetic background of Asian–Oceania populations, the consensus recommends the use of local population reference values for indicators such as BMI, waist circumference (WC), VFA, and BFP. Regarding muscle mass measurement, the recommended index is consistent with the consensus of the Asian Working Group for Sarcopenia (AWGS2019), namely, ASMI. The ASMI eliminates the impact of height on absolute muscle mass, making muscle mass assessments more comparable across individuals of different heights, and it has been validated in multiple studies within Asian populations [62].

Additionally, this consensus innovatively introduces the concept of “possible sarcopenic obesity,” defined as meeting obesity criteria alongside reduced muscle strength or physical function (with normal or undetected muscle mass). Since declines in muscle strength or physical function may precede reductions in muscle mass, their impact on health may be more pronounced than the loss of muscle mass itself [52]. The concept of “possible sarcopenic obesity” facilitates early intervention for this population to reduce disease progression risks. Similarly, scholars previously proposed the concept of dynapenic obesity (DO). DO is characterized by the simultaneous presence of dynapenia (weakness and/or slowness) and excess adiposity [63]. It is evident that the concept of possible sarcopenic obesity closely resembles that of dynapenic obesity, both emphasizing the health hazards of combined muscle strength or physical function decline with obesity. However, current research on sarcopenic obesity tends to focus more on muscle mass loss rather than the integrated performance of muscle mass, grip strength, and physical function. The concepts of possible sarcopenic obesity and dynapenic obesity facilitate our attention to changes in grip strength and physical function before alterations in muscle mass occur.

### 5.4. Regional Applicability of Diagnostic Criteria

The diagnostic criteria for SO require further validation in different regions. For example, a study applying the ESPEN/EASO criteria to elderly rehabilitation patients in Japan reported that the prevalence of SO was only 4.3–5.3% and was not significantly associated with adverse functional outcomes. These findings suggest that the ESPEN/EASO criteria may not be suitable for the elderly Asian population, highlighting the need to optimize diagnostic standards on the basis of local conditions [64].

### 5.5. Advantages and Disadvantages of Muscle Mass Measurement Devices

Currently, the devices used to assess muscle mass each have their own characteristics, and their clinical application requires selection on the basis of the specific context.

Dual-energy X-ray absorptiometry (DXA): DXA has high sensitivity and is commonly used in clinical practice, but the accuracy of measurements may be affected by factors such as the proportion of skeletal muscle in total lean body mass, tissue thickness, and water content [1].

Bioelectrical impedance analysis (BIA): This device is lightweight, easy to operate, and can quickly assess the distribution of muscle and fat in limbs, trunk, and other body parts, making it suitable for primary healthcare settings. However, BIA does not directly measure body composition but estimates it through the electrical conductivity of muscle and fat [65]. As a result, a high body fat percentage or changes in the patient’s fluid balance may interfere with its accuracy [65,66].

Computed Tomography (CT): CT is considered one of the gold standards for muscle mass measurement and is especially useful for patients with comorbidities who also need CT scans [67]. ESPEN/EASO recommends assessing muscle mass at the level of the third lumbar vertebra, as it strongly correlates with total body muscle mass [1]. However, CT is associated with high costs and exposure to X-rays, which limits its widespread clinical use. Additionally, sex differences should be considered: men tend to accumulate fat in the visceral area (apple-shaped body), whereas women typically store fat subcutaneously (pear-shaped body), requiring different regions to be selected for muscle mass evaluation on the basis of sex [68].

Magnetic Resonance Imaging (MRI): This is the gold standard for muscle mass measurement [67]. It can accurately measure whole-body muscle mass and the degree of fat infiltration [69]. However, owing to its high cost, MRI is used primarily in research settings.

In summary, the core challenge in the diagnosis of SO lies in the unification of standards and regional adaptability, while the selection of measurement devices needs to balance accuracy, convenience, and cost. Future multicenter studies are needed to optimize the diagnostic system and provide reliable evidence for the early identification and intervention of SO.

## 6. Association Between Sarcopenic Obesity and Atherosclerosis

The association between SO and atherosclerosis has recently become a research hotspot. Existing evidence covers lesions in specific sites, such as the carotid and coronary arteries, as well as multiple dimensions, including arterial stiffness and long-term cardiovascular risk. Most studies support an association between the two, with visceral fat-related indicators (such as VFA and BFP) and the ASM/VFA ratio showing significant correlations. However, the conclusions exhibit heterogeneity due to factors such as diagnostic criteria and study populations. The following section reviews the characteristics and controversies of the associations between SO and atherosclerosis from four perspectives.

### 6.1. Sarcopenic Obesity and Carotid Atherosclerosis

Carotid atherosclerosis is typically assessed via carotid intima-media thickness (cIMT) measured via carotid ultrasound [70]. Although different diagnostic criteria for SO and various comorbidities have been used across studies, most research suggests that SO is associated with the occurrence and severity of carotid atherosclerosis (Table 2).

Milic et al. (2023) reported that in HIV-infected individuals, low grip strength combined with high VFA increased the risk of carotid atherosclerosis by 5% (IRR = 1.05, 95% CI 1.01–1.09), which was greater than the risk associated with sarcopenia or obesity alone [71]. Luo et al. (2025) reported that SO was closely associated with endothelial dysfunction (OR = 1.575, 95% CI 1.017–2.441) and carotid atherosclerosis (OR = 1.430, 95% CI 1.022–2.001) [72]. In patients with type 2 diabetes (T2DM), Shin et al. (2021) reported that a decrease in appendicular skeletal muscle mass adjusted for BMI (ASM/BMI) increased the risk of high cIMT by 51% (OR = 1.51, 95% CI 1.03–2.22) and increased the risk of a high plaque score by 77% (OR = 1.77, 95% CI 1.23–2.56). In this study, the grip strength/BMI ratio also showed a similar association [73]. Similarly, Bouchi et al. (2016) also found in patients with type 2 diabetes that an elevated android fat/gynoid fat ratio (A/G ratio) combined with low Skeletal Muscle Index (SMI) was associated with carotid atherosclerosis (β = 0.408, *p* = 0.01) [74]. They further demonstrated that an elevated A/G ratio was not associated with carotid atherosclerosis in the general population or in individuals with high SMI, highlighting the importance of simultaneously assessing muscle and fat composition [74]. Oh et al. (2011) found that the ratio of abdominal visceral thickness to thigh muscle thickness was associated with cIMT thickening (β = 0.312) [75]. Nakano et al. (2017) reported that SO defined by VFA (SO_VFA_) was associated with significantly greater carotid atherosclerosis than was isolated obesity, and the maximum cIMT (cIMTmax) might inversely affect the occurrence of SO (OR = 2.40) [76], suggesting a potential bidirectional relationship between the two.

There is still controversy regarding whether sarcopenia or obesity plays a more significant role in the relationship between SO and carotid atherosclerosis.

Some studies suggest that sarcopenia has a greater impact. Sato et al. (2024) found that in elderly diabetic patients, the association of the SO_VFA_ with carotid atherosclerosis (β = 0.384) was stronger than that of the SO defined by the BFP (SO_BFP_, β = 0.237), and muscle loss was a necessary condition for the association between the VFA and cIMT (VFA was positively correlated with IMTmax only in the sarcopenia subgroup) [77]. Lu et al. (2023) reported that a low ASMI significantly increased the risk of carotid plaque in postmenopausal women (OR = 2.43–4.82, *p* < 0.001), independent of BMI [78]. Furthermore, Cao et al. (2020) reported that in patients with metabolic syndrome and a waist-to-height ratio ≥ 0.5, muscle mass loss was an important risk factor for carotid atherosclerosis (OR = 2.788, 95% CI: 1.559–4.985), with greater muscle mass offering protective effects against atherosclerosis [79].

Some studies indicate that obesity has a greater impact on carotid atherosclerosis. Kang et al. (2021) reported that low skeletal muscle mass (LSMM) was an important factor influencing carotid atherosclerosis in patients with nonalcoholic fatty liver disease (NAFLD) (OR = 2.05–2.95). Subgroup analysis revealed that the risk was greater in the obese (BMI ≥ 25 kg/m^2^) subgroup (OR = 2.44–3.54). However, in nonobese (BMI < 25 kg/m^2^) NAFLD patients, LSMM was not significantly associated with carotid atherosclerosis, suggesting that obesity may play a key role [80].

However, conflicting evidence exists regarding the association between SO and atherosclerosis. Xia et al. (2021) reported no significant association between SO_BMI_ (SO defined by BMI) and carotid atherosclerosis (OR = 0.97, 95% CI 0.57–1.63) [81]. Heo et al. (2018) reported that low appendicular skeletal muscle mass adjusted for weight (ASM/Wt) was associated with higher cIMT (OR = 2.78, 95% CI 1.09–7.13) and plaque risk (OR = 2.48, 95% CI 1.04–5.90) only in nonobese men (BMI < 25 kg/m^2^) but not in obese men or women, regardless of BMI [82]. Cho et al. (2023) reported that sarcopenia significantly increased the risk of carotid plaque progression in NAFLD patients with T2DM (AOR = 2.20, 95% CI 1.21–4.00), particularly in subgroups including women, nonobese individuals (BMI < 25 kg/m^2^), and those without central obesity [83]. Both Heo and Cho’s studies suggest associations primarily in nonobese populations, which do not specifically support a direct link between SO and atherosclerosis. Additionally, Kim et al. (2019) reported no association between SO_WC_ (SO defined by WC) and cIMT in newly diagnosed T2DM patients [84].

In summary, most studies have shown an association between SO and carotid atherosclerosis, with both muscle mass loss and obesity independently or synergistically contributing to the development of lesions. However, the lack of standardized diagnostic criteria for SO, coupled with variations in the diagnostic indices used by researchers, is likely the primary factor influencing the study outcomes. Additionally, other factors that may affect the conclusions include the sample size and the presence of comorbidities (such as T2DM, NAFLD, HIV, etc.) in the study populations.

### 6.2. Sarcopenic Obesity and Coronary Artery Atherosclerosis

The severity of coronary atherosclerosis can be assessed via the coronary calcium score (CT) or the Gensini score (angiography). Existing studies consistently support that SO promotes coronary atherosclerosis (Table 2), and visceral fat-related indicators may demonstrate superior predictive value. Deposition of epicardial adipose tissue (EAT) may represent a key mechanism mediating the association between SO and coronary atherosclerosis [85].

Jun et al. (2021) reported that SO_WC_ increased the risk of coronary artery calcification by 116% (OR = 2.16, 95% CI 1.98–2.36) and the risk of progression by 54% (HR = 1.54, 95% CI 1.37–1.75), with a greater risk than sarcopenia or abdominal obesity alone [86]. Ren’s (2025) meta-analysis indicated that SO is significantly associated with coronary artery disease (OR = 3.40, 95% CI 1.96–5.91) [87]. Wang (2023) further confirmed that a decrease in muscle mass (β = −31.925, *p* < 0.001) and an increase in waist circumference (β = 4.819, *p* < 0.001) in SO patients are independently associated with the degree of coronary artery stenosis in coronary heart disease (CHD) patients [88].

Milic et al. (2023) reported that in HIV-infected individuals, a low ASMI combined with a high VFA increased the risk of coronary artery calcification (IRR = 1.03, 95% CI 1.01–1.05), whereas no such association was found when a low ASMI was combined with a high BMI [71]. These findings demonstrated that the VFA may better reflect the impact of SO on coronary arteries than BMI. In contrast, Chung et al. (2021) found that the SO_BMI_ was independently associated with coronary artery calcification (OR = 1.92, 95% CI 1.16–3.18) [89], but this study did not compare the effects of the VFA and BMI.

### 6.3. Sarcopenic Obesity and Arterial Stiffness

The brachial-ankle pulse wave velocity (baPWV) is a commonly used indicator for assessing large artery stiffness and can assist in diagnosing peripheral and central artery stenosis [90,91]. An elevated baPWV reflects a worsening degree of arterial sclerosis and significantly increases the risk of cardiovascular and cerebrovascular diseases [92,93]. The cardio-ankle vascular index (CAVI), developed in Japan, is used to assess the function and stiffness of the arteries from the aorta to the ankle [94]. Prospective studies in Asian populations have indicated that a CAVI ≥ 9.5 significantly increases the risk of cardiovascular events and all-cause mortality [95]. Several studies have confirmed an association between SO and elevated baPWV and CAVI (Table 2).

Multiple studies have shown that the SOVFA or the ratio of ASM/VFA is closely related to arterial stiffness, highlighting the importance of joint assessment of muscle mass and VFA. Luo et al. (2025) reported a strong association between the SOVFA and arterial stiffness (OR = 1.382, 95% CI 1.050–1.818) [72]. Ohara et al. (2014) observed that the baPWV was significantly greater in the SOVFA group than in the sarcopenia or obesity-only groups [96]. Kim et al. (2011) reported that the ASM/VFA was an independent negative predictor of arterial stiffness (β = −59.505, *p* = 0.002, R^2^ = 0.57), whereas the correlation with the ASMI was weaker [97]. The authors suggested that the ASMI is more susceptible to interference from factors such as BMI, waist circumference, and triglycerides, whereas the ASM/VFA better reflects the muscle-fat balance [97]. Xu et al. (2018) similarly found in their study of type 2 diabetes patients that ASM/VFA demonstrated superior predictive value for arterial stiffness compared to ASM, VFA, or ASM/height^2^ alone [98]. Matsumoto et al. (2022) further reported that high VFA combined with reduced physical function was also independently associated with arterial stiffness (β = 48, *p* = 0.01) [99].

Interestingly, Fantin et al. (2024) reported that both sarcopenia and sarcopenic obesity are associated with increased arterial stiffness. SO showed a stronger association with central arterial stiffness (assessed by cfPWV, carotid–femoral pulse wave velocity) (β = 3.08, *p* = 0.031, R^2^ = 0.20), whereas sarcopenia was more significantly associated with peripheral arterial stiffness (assessed by CAVI) (β = 1.38, *p* = 0.042, R^2^ = 0.12) [100]. This suggests that SO may exert a greater impact on central arteries.

In addition to VFA, obesity indicators such as BMI, WC, and BFP have also garnered attention. Bak et al. (2024) reported that SO, defined by a combination of BMI and WC, was associated with arterial stiffness risk (OR = 2.40, 95% CI 1.07–5.38) [47]. Wang et al. (2020) reported that SOBFP was an important determinant of CAVI (OR = 11.87, 95% CI 1.80–235.22), whereas the effects of isolated obesity or muscle mass reduction were not significant [101]. Interestingly, this study also revealed the “obesity paradox,” where the BMI and weight in the arterial sclerosis group were lower than those in the non-arterial sclerosis group [101]. This may be because BMI and weight gain do not adequately reflect changes in body composition and fat distribution. Liu et al. (2025) focused on the Skeletal Muscle Fat Index (SMFI) in their study, which reflects the degree of muscle fat infiltration. Their research demonstrated that the combination of SMI and SMFI outperformed either SMI or SMFI alone in predicting arterial stiffness [102].

However, the association between SO and arterial stiffness shows sex differences and heterogeneity across studies. Jang et al. (2023) reported that in men, the SOVFA was an important factor for increased baPWV, whereas no such association was observed in women, possibly due to confounding factors, such as age [103]. Kim et al. (2019) reported no association between SOWC and baPWV in newly diagnosed T2DM patients [84]. This may be related to the early stage of diabetes, where arterial stiffness has not yet manifested, and long-term follow-up is needed for verification. The discrepancies in conclusions are primarily due to differences in study population characteristics (such as comorbidities) and the SO diagnostic criteria.

### 6.4. Sarcopenic Obesity and 10-Year ASCVD Risk

The 10-year ASCVD risk score is widely used internationally and is based on the 2013 American College of Cardiology/American Heart Association (ACC/AHA) assessment model [104]. In 2016, the China-PAR model was developed by the National Center for Cardiovascular Diseases in China and is specifically tailored for the Chinese population [105]. Most studies indicate that SO is associated with increased 10-year ASCVD risk (Table 2).

Han et al. (2024) reported that low ASM/VFA combined with low ASM/BMI increases the 10-year ASCVD risk by 3.15 times (OR = 4.15, 95% CI 1.65–10.45), with this synergistic effect being stronger than a single measure [106]. Although ASM/VFA was not associated with 10-year ASCVD risk in this study, the innovative finding was the significant association of low ASM/VFA combined with low ASM/BMI. Additionally, four other studies, which used different criteria, confirmed the association between SO and 10-year ASCVD risk in specific populations. Chen et al. (2025) reported that an increased total fat-to-total muscle ratio (FMR) significantly elevated the 10-year ASCVD risk in hypertensive patients (OR = 3.102, 95% CI 2.055–4.682) [107]. Chien et al. (2025) reported that patients with metabolic dysfunction-associated fatty liver disease (MASLD) and SO_BFP_ had a greater number of individuals at high risk for 10-year ASCVD than non-SO_BFP_ patients did (10.33% vs. 7.12%) [108]. Yan et al. (2024) reported that SO_BFP_ was a significant factor for 10-year ASCVD risk in patients with hypertension (OR = 2.55, 95% CI 1.07–6.11) and NAFLD (OR = 6.57, 95% CI 2.20–19.65) [109]. Leem et al. (2022) reported that a decrease in ASM/BMI significantly increased the 10-year ASCVD risk in male COPD patients (OR = 2.32, 95% CI 1.05–5.15) [110].

**Table 2:** Main characteristics and outcomes of selected clinical observational studies linking sarcopenic obesity and atherosclerosis.

First Author (Year) Origin [Reference]	Study Type	Characteristics of Individuals (*n*)	Diagnosis of Sarcopenic Obesity		Atherosclerosis Assessment	Main Findings
			Sarcopenia	Obesity		
Shi et al. (2024) China [34]	Cross-sectional	Hospitalized patients ≥ 45 years (469)	ASMI; VFA; Grip strength; SVR (ASMI/VFA)		The 10-year ASCVD risk assessment model from China-PAR	In elderly women, low ASMI, low grip strength, and low SVR significantly increase 10-year ASCVD risk. In elderly men, only decreased grip strength increases 10-year ASCVD risk.
Bak et al. (2024) Korea [47]	Cross-sectional	Adults ≥ 40 years old undergoing health examination (20,601)	BIA: ASMI (M < 7.0 kg/m^2^, F < 5.7 kg/m^2^)	BMI ≥ 25 kg/m^2^; WC (M ≥ 90 cm, F ≥ 85 cm)	baPWV > 1800 cm/s	SO is associated with arterial stiffness risk, with muscle mass reduction potentially playing a greater role.
Milic et al. (2023) Italy [71]	Retrospective cohort (follow-up: 8 years)	HIV-infected patients (2379)	Grip strength below U.S. population thresholds or DXA: ASMI (M < 7.26 kg/m^2^, F < 5.45 kg/m^2^) or SPPB < 11	BMI ≥ 30 kg/m^2^ or DXA/CT: VFA ≥ 160 cm^2^	US: cIMT ≥ 1 mm or presence of carotid plaque. CT: Coronary artery calcification score calculated by the Agatston method > 10	Low grip strength combined with high VFA significantly increased the risk of carotid atherosclerosis, higher than sarcopenia or obesity alone. Low ASMI combined with high VFA significantly increased the risk of coronary artery calcification.
Luo et al.(2025) China [72]	Cross-sectional	Adults ≥ 50 years old undergoing health examination (1010)	BIA: Skeletal muscle mass to body weight ratio (M < 39.3%, F < 33.9%)	BIA: VFA ≥ 100 cm^2^	US: cIMT ≥ 1.5 mm or localized carotid thickening ≥ 50%; Arterial stiffness: baPWV > 1800 cm/s; Endothelial dysfunction: FMD < 10%)	SO is significantly associated with endothelial dysfunction, carotid atherosclerosis, and arterial stiffness.
Shin et al. (2021) Korea [73]	Cross-sectional	T2DM patients > 30 years old (1185)	DXA: ASM/BMI, Grip strength/BMI		US: High cIMT (M ≥ 0.970 mm, F ≥ 0.895 mm); Plaque score ≥ 3	Decrease in ASM/BMI and grip strength/BMI is associated with high cIMT and high plaque score.
Bouchi et al. (2016) Japan [74]	Cross-sectional	T2DM patients (259)	DXA: SMI (M < 7.0 kg/m^2^, F < 5.4 kg/m^2^);	DXA: android fat/gynoid fat ratio (A/G ratio)	US: cIMT	Elevated A/G ratio combined with low SMI is associated with carotid atherosclerosis.
Oh et al.(2011) Korea [75]	Cross-sectional	Newly diagnosed T2DM males (68); Healthy males (15)	US: Abdominal visceral thickness; CT: Thigh muscle thickness		US: cIMT	Thigh muscle thickness is correlated with cIMT thickening.
Nakano et al.(2017) Japan [76]	Cross-sectional	T2DM patients with obesity aged ≥ 65 years (55)	CT: Cross-sectional area of the psoas muscle at the L3 vertebral level, normalized as TPA < 500 mm^2^/m^2^	CT: VFA > 100 cm^2^	US: cIMTmax	SO exhibits more severe carotid atherosclerosis compared to obesity alone, and cIMTmax may be associated with the development of SO.
Sato et al. (2024) Japan [77]	Cross-sectional	Hospitalized patients ≥ 75 years old, 94.9% had T2DM (118)	BIA: SMI (M < 7.0 kg/m^2^, F < 5.7 kg/m^2^); Grip strength (M < 28 kg, F < 18 kg)	BIA: BFP (M ≥ 29%, F ≥ 38.4%); CT: VFA ≥ 100 cm^2^.	US: IMTmax; Pulse Wave Analyzer: ABImin	SO is a significant influencing factor for carotid atherosclerosis in elderly T2DM patients (VFA was superior to BFP). The positive correlation between VFA and IMTmax was only significant in the sarcopenia subgroup.
Lu et al. (2023) China [78]	Cross-sectional	Postmenopausal women aged 40–88 years (2048)	BIA: ASMI ≤ 5.7 kg/m^2^	BMI ≥ 24 kg/m^2^	US: cIMT ≥ 1.0 mm or localized carotid thickening ≥ 50%	Decreased ASMI significantly increased carotid plaque risk in postmenopausal women, independent of BMI categories.
Cao et al.(2020) China [79]	Cross-sectional	Aged 24–85 years, MetS (1950), non-MetS (184)	Derived by anthropometric formula: ASM, ASM/Wt × 100% (< −1 SD)	WHTR ≥ 0.5	US: cIMT ≥ 1.0 mm	Decreased muscle mass is a significant influencing factor for carotid atherosclerosis in MetS patients (with WHTR ≥ 0.5). Muscle mass exerts a protective effect against atherosclerosis.
Kang et al. (2021) Korea [80]	Cross-sectional	NAFLD patients (683)	BIA: ASM/BMI (M < 0.789, F < 0.512)	BMI ≥ 25 kg/m^2^	US: cIMT ≥ 1.0 mm or localized carotid thickening ≥ 50%	ASM/BMI is a significant influencing factor for subclinical atherosclerosis in NAFLD patients, and obesity may exert a stronger effect than decreased muscle mass.
Xia et al. (2021) China [81]	Cross-sectional	Community-dwelling adults ≥ 45 years old (2432)	DXA: ASMI (M < 7.0 kg/m^2^, F < 5.4 kg/m^2^)	BMI ≥ 24 kg/m^2^	US: cIMT ≥ 1.1 mm	SO showed no significant association with carotid atherosclerosis.
Heo et al. (2018) Korea [82]	Cross-sectional	Community-dwelling adults aged 30–64 years (1869)	BIA: ASM/Wt	BMI ≥ 25 kg/m^2^	US: High cIMT (M ≥ 0.755 mm, F ≥ 0.724 mm); cIMT ≥ 1.0 mm or localized carotid thickening ≥ 50%	The association between reduced ASM/Wt and carotid atherosclerosis was observed only in nonobese males.
Cho et al. (2023) Korea [83]	Prospective cohort (follow-up: 6–8 years)	Patients with T2DM aged ≥ 19 years (852)	BIA: SMI (< −2SD) or ASMI (M < 7.0 kg/m^2^, F < 5.7 kg/m^2^)	BMI ≥ 25 kg/m^2^ or WC (M ≥ 90 cm, F ≥ 85 cm)	US: Carotid plaque progression (cIMT ≥ 1.5 mm or plaque protruding ≥ 50% into the lumen or a distinct hyperechoic area).	NAFLD combined with sarcopenia significantly increased the risk of carotid plaque progression in T2DM patients. The risk of plaque progression was even higher in women and in those with normal BMI and WC who also had sarcopenia.
Kim et al. (2019) Korea [84]	Cross-sectional	Newly diagnosed, drug-naive T2DM patients (233)	DXA: ASMI (M < 7 kg/m^2^, F < 5.4 kg/m^2^)	WC (M ≥ 90 cm, F ≥ 85 cm)	US: cIMT > 0.9 mm or presence of plaque; baPWV > 1800 cm/s	SO showed no association with cIMT or baPWV.
Jun et al. (2021) Korea [86]	Cross-sectional & Retrospective cohort (follow-up: 8 years)	Adults ≥ 20 years old undergoing health examination (Cross-sectional:19,728, Prospective cohort:5401)	BIA: SMI ≤ −1.0 SD	WC (M ≥ 90 cm, F ≥ 85 cm	CT: Coronary artery calcification (CAC score > 0); CAC progression (The square root of the difference in CAC scores between follow-up and baseline periods was greater than 2.5)	SO increases the risk of CAC incidence and progression, with higher risk than sarcopenia or isolated abdominal obesity alone.
Ren (2025) China [87]	Meta-analysis	Community-dwelling older adults from multiple countries (42,683)	DXA/BIA/CT: Muscle mass; Grip strength; Physical function	BMI; WC; BFP	Clinically diagnosed CHD	SO is a significant risk factor for coronary artery disease.
Wang (2023) China [88]	Cross-sectional	CHD patients ≥ 60 years (127)	BIA: SMI (M < 7.0 kg/m^2^, F < 5.7 kg/m^2^; Grip strength (M < 26 kg, F < 18 kg); Gait speed < 0.8 m/s	WC (M ≥ 102 cm, F ≥ 88 cm)	Coronary angiography: Gensini score > 40	Both decreased muscle mass and increased waist circumference in SO patients correlate with coronary stenosis severity in CHD patients.
Chung et al. (2021) Korea [89]	Cross-sectional	Health examinees (1282)	BIA: ASM% < −2SD	BMI ≥ 25 kg/m^2^	CT: CAC score ≥ 100	SO is independently associated with CAC, and confers significantly higher risk than isolated obesity or isolated sarcopenia.
Ohara et al. (2014) Japan [96]	Cross-sectional	Middle-aged and elderly individuals without history of cardiovascular events (1470)	CT: Thigh muscle cross-sectional area (CSA) < −1 SD or < 20th percentile of the study population	CT: VFA > 100 cm^2^	baPWV > 1800 cm/s	SO shows a stronger correlation with arterial stiffness than isolated sarcopenia or isolated obesity.
Kim et al. (2011) Korea [97]	Cross-sectional	Healthy adults aged 20–80 years (510)	DXA/CT: MFR (ASM/VFA); DXA: ASMI		baPWV > 1800 cm/s	MFR may be an independent negative predictor of arterial stiffness, whereas ASMI shows a weaker correlation with arterial stiffness.
Xu et al. (2018) China [98]	Cross-sectional	T2DM patients (423)	BIA: ASM (g)/VFA (cm^2^), divide into tertiles		baPWV > 1800 mm/s	Compared to ASM, VFA, or ASM/height^2^ alone, ASM/VFA demonstrates greater predictive value for arterial stiffness.
Matsumoto et al. (2022) Japan [99]	Cross-sectional	Adults ≥ 50 years old undergoing health examination (137)	Two-step test; Stand-up test	CT: VFA > 100 cm^2^	baPWV	High VFA combined with reduced physical function is also independently associated with arterial stiffness.
Fantin et al. (2024) Italy [100]	Cross-sectional	Geriatric Inpatients (77)	BIA: SMI (M < 10.75 kg/m^2^, F < 6.75 kg/m^2^; Grip strength (M < 30 kg, F < 20 kg)	BMI ≥ 30 kg/m^2^; WC (M ≥ 102 cm, F ≥ 88 cm)	cfPWV; CAVI	Sarcopenia and SO are both associated with increased arterial stiffness, and SO may exert a greater effect on the central arteries.
Wang et al. (2020) China [101]	Cross-sectional	Hospitalized elderly patients (1150)	BIA: ASMI (M < 7 kg/m^2^, F < 5.7 kg/m^2^)	BIA: BFP (M ≥ 25%,F ≥ 35%); BIA: Visceral fat mass/Total fat mass ratio	CAVI ≥ 9 m/s	SO is a significant influencing factor for CAVI, whereas isolated obesity or reduced muscle mass show no significant effects.
Liu et al. (2025) China [102]	Prospective cohort (follow-up: 3 years)	Inpatients ≥ 18 years old	CT: Measure the paraspinal muscle area at the T12 level and calculate SMI and SMFI (muscle area (cm^2^)/muscle density (HU))		baPWV ≥ 1400 cm/s	SMI combined with SMFI outperforms either SMI or SMFI alone in predicting arterial stiffness.
Jang et al.(2023) Korea [103]	Cross-sectional	Health examination adults ≥ 21 years (7177)	BIA: ASMI (M < 7 kg/m^2^, F < 5.7 kg/m^2^)	BIA: VFA > 100 cm^2^, Visceral fat mass/Total fat mass ratio	baPWV > 1800 cm/s	In males, SO is a significant risk factor for elevated baPWV. Decreased ASMI may contribute more to arteriosclerosis than VFA. In females, no association was found between SO and baPWV.
Han et al. (2024) Korea [106]	Cross-sectional	Adults ≥ 20 years at high risk for NAFLD (1455)	BIA: ASM/VFA (<sex-specific tertile); ASM/BMI (M < 0.789, F < 0.512)		The 10-year ASCVD risk assessment model from ACC/AHA	Low ASM/VFA combined with low ASM/BMI synergistically increases 10-year ASCVD risk, whereas isolated low ASM/VFA or isolated low ASM/BMI shows no significant association with 10-year ASCVD risk.
Chen et al. (2025) China [107]	Cross-sectional	Adults ≥ 40 years undergoing health examination (1592)	BIA: FMR		The 10-year ASCVD risk assessment model from ACC/AHA	Elevated FMR significantly increases 10-year ASCVD risk in hypertensive patients.
Chien et al. (2025) Taiwan, China [108]	Cross-sectional	MASLD patients (223)	DXA: ASM% (M < 30.8%, F < 24.3%)	DXA: BFP (M > 25%, F > 38%)	The 10-year ASCVD risk assessment model from ACC/AHA	MASLD with SO had more individuals at high 10-year ASCVD risk than non-SO patients.
Yan et al.(2024) China [109]	Cross-sectional	Hypertensive patients (1512); NAFLD patients (1249)	BIA: ASMI (M ≤ 7.0 kg/m^2^, F ≤ 5.7 kg/m^2^)	BIA: BFP (M > 25%, F > 35%)	The 10-year ASCVD risk assessment model from ACC/AHA	SO is a significant risk factor for 10-year ASCVD in hypertensive and NAFLD patients.
Leem et al. (2022) Korea [110]	Cross-sectional, (data from KNHANES 2008–2011)	Male COPD patients (704)	DXA: ASM/BMI < −1SD	WC ≥ 90 cm; BMI ≥ 25 kg/m^2^; FMI ≥ 7.0 kg/m^2^	The 10-year ASCVD risk assessment model from ACC/AHA	Decreased ASM/BMI significantly increases 10-year ASCVD risk in male COPD patients, independent of BMI, WC, and FMI (fat mass/height^2^).

Abbreviations: ABImin, Minimum Ankle-Brachial Index; ACC/AHA, American College of Cardiology/American Heart Association; ASCVD, Atherosclerotic Cardiovascular Disease; ASM%, ASM/Body Weight × 100%; ASM, Appendicular Skeletal Muscle Mass; ASMI, Appendicular Skeletal Muscle Index (ASM/height^2^); baPWV, Brachial-Ankle Pulse Wave Velocity; BFP, Body Fat Percentage; BIA, Bioelectrical Impedance Analysis; BMI, Body Mass Index; CAC, Coronary Artery Calcium; CAVI, Cardio-Ankle Vascular Index; cfPWV, carotid–femoral pulse wave velocity; CHD, Coronary Heart Disease; China-PAR, Chinese ASCVD Risk Prediction Model; cIMT, Carotid Intima-Media Thickness; cIMTmax, Maximum Carotid Intima-Media Thickness; COPD, Chronic Obstructive Pulmonary Disease; CSA, Cross-Sectional Area; CT, Computed Tomography; DXA, Dual-Energy X-ray Absorptiometry; F, Female; FMD, Flow-Mediated Dilatation; FMI, Fat Mass Index (fat mass/height^2^); FMR, Fat-to-Muscle Ratio (Total body fat mass/Total body muscle mass); Gensini score, Quantitative coronary stenosis scoring system; L3, Third Lumbar Vertebra; M, Male; MASLD, Metabolic Dysfunction-Associated Steatotic Liver Disease; MFR, Muscle-to-Fat Ratio (ASM/VFA); NAFLD, Non-Alcoholic Fatty Liver Disease; SD, Standard Deviation; SMI, Skeletal Muscle Index; SO, Sarcopenic Obesity; SPPB, Short Physical Performance Battery; SVR, Skeletal Muscle to Visceral Fat Ratio (ASMI/VFA); T2DM, Type 2 Diabetes Mellitus; TPA, Total Psoas Area; US, Ultrasound; VFA, Visceral Fat Area; WC, Waist Circumference; WHTR, Waist-to-Height Ratio; Wt, Body Weight.

However, Shi et al. (2024) reported that the association between SO and 10-year ASCVD risk may exhibit sex differences. In women over 65 years of age, a low ASMI (OR = 0.109, 95% CI 0.038–0.317), low grip strength (OR = 0.899, 95% CI 0.824–0.981), or a low ASMI/VFA ratio (OR = 0.042, 95% CI 0.013–0.140) significantly increased the 10-year ASCVD risk. In men over 65 years of age, however, only a decrease in grip strength (OR = 0.910, 95% CI 0.845–0.980) was an influencing factor [34]. These differences may be related to variations in sex hormone levels and body composition changes between men and women.

## 7. Pathophysiological Mechanisms Between Sarcopenic Obesity and Atherosclerosis

**Figure 1:**
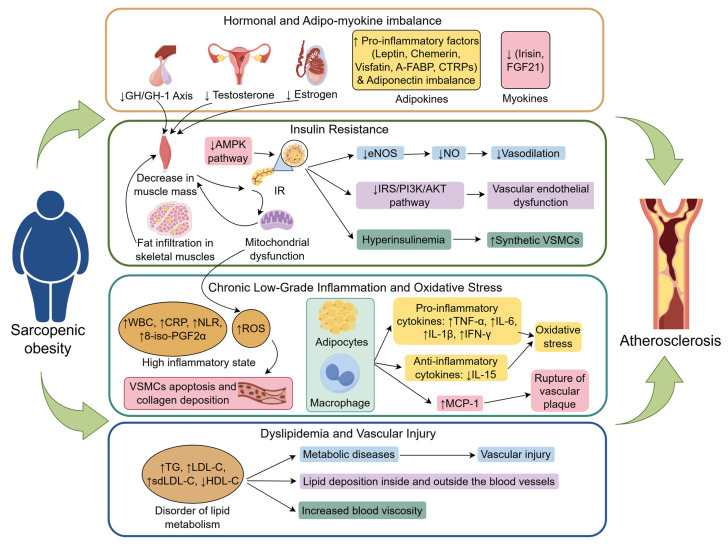
The mechanism that sarcopenic obesity promotes atherosclerosis (created with figdraw.com). Sarcopenic obesity promotes the development and progression of atherosclerosis through multiple interconnected mechanisms, including hormonal imbalances, dysregulation of adipo-myokines, insulin resistance, chronic low-grade inflammation, oxidative stress, and disturbances in lipid metabolism. Abbreviations: Adipo-myokine, Adipokines and myokines; A-FABP, Adipocyte Fatty Acid-Binding Protein; AMPK pathway, Adenosine Monophosphate-Activated Protein Kinase pathway; CRP, C-Reactive Protein; CTRPs, C1q/TNF-related Proteins; eNOS, Endothelial Nitric Oxide Synthase; FGF21, Fibroblast Growth Factor 21; GH/IGF-1 Axis, Growth Hormone/Insulin-like Growth Factor-1 Axis; HDL-C, High-Density Lipoprotein Cholesterol; IFN-γ, Interferon-gamma; IL-15, Interleukin-15; IL-1β, Interleukin-1 beta; IL-6, Interleukin-6; IR, Insulin Resistance; IRS/PI3K/AKT pathway, Insulin Receptor Substrate/Phosphoinositide 3-Kinase/Protein Kinase B Pathway; LDL-C, Low-Density Lipoprotein Cholesterol; MCP-1, Monocyte Chemoattractant Protein-1; NLR, Neutrophil-to-Lymphocyte Ratio; NO, Nitric Oxide; oxLDL-C, Oxidized Low-Density Lipoprotein Cholesterol; ROS, Reactive Oxygen Species; TG, Triglycerides; TNF-α, Tumor Necrosis Factor-alpha; VSMCs, Vascular Smooth Muscle Cells; 8-iso-PGF2α, 8-isoprostaglandin F2α; ↑, Increased expression or enhanced effect; ↓, expression reduction or diminished effect; Other arrows, Pathogenic mechanism of the next step.

SO is not simply the overlap of sarcopenia and obesity. Instead, it creates a “pathological network” that promotes atherosclerosis through the interaction of multiple systems, including hormonal and muscle-fat factor imbalances, insulin resistance, chronic inflammation, and lipid metabolism disturbances (Figure 1). The following section explores the mechanistic link between SO and atherosclerosis through four core pathways.

### 7.1. Hormonal and Adipo-Myokine Imbalance

During aging, changes in the levels of key hormones, such as testosterone, estrogen, and growth hormone, not only regulate SO but also contribute to the development of atherosclerosis. Specifically, a decrease in the growth hormone/insulin-like growth factor-1 (GH/IGF-1) axis impairs muscle synthesis via the PI3K-AKT/PKB-mTOR signaling pathway, leading to muscle atrophy [86]. Moreover, GH/IGF-1 axis dysfunction promotes atherosclerosis by affecting lipid metabolism and inhibiting vascular relaxation [111]. A decrease in testosterone levels in men directly weakens the ability of testosterone to stimulate skeletal muscle protein synthesis. Obesity further accelerates this process by increasing the activity of aromatase in adipose tissue, which enhances the conversion of testosterone to estradiol, further lowering testosterone levels [112,113]. Testosterone deficiency, in turn, reduces vascular relaxation and promotes vascular stiffness by increasing matrix metalloproteinase-2 (MMP-2) in arterial smooth muscle cells, which degrades the elastic matrix [114]. In women, estrogen has a protective effect on cardiovascular health, and a decline in sex hormones (especially after menopause) accelerates muscle loss, fat redistribution, and atherosclerosis [115]. With aging, a decrease in testosterone, estrogen, and growth hormone contributes to increased insulin resistance by reducing muscle mass, thereby creating a vicious cycle of hormone-insulin resistance interactions [116,117,118].

In SO patients, dysregulation of adipo-myokines accelerates the progression of atherosclerosis. Proinflammatory adipokines, such as leptin, chemokines, visfatin, adipocyte fatty acid-binding protein (A-FABP), and C1q/TNF-related proteins (CTRPs), which activate macrophages and induce endoplasmic reticulum stress and vascular inflammatory responses, are elevated in SO patients [119,120,121]. Additionally, an imbalance in adiponectin (an adipokine) levels, specifically a reduction in the proportion of high-molecular-weight adiponectin (HMW-Adiponectin) to total adiponectin, impairs insulin sensitivity and inhibits vascular relaxation, promoting arterial stiffness [114,122]. Furthermore, dysregulation of myokines in SO patients—characterized by diminished secretion of irisin and FGF21 or heightened secretion of myostatin (MSTN)—can exacerbate insulin resistance, diminish antioxidant capacity, accelerate LDL oxidation, and provoke the release of proinflammatory factors from adipose tissue. This ultimately undermines the protective roles of myokines in glucose homeostasis and the vascular endothelium [71,103,123,124].

### 7.2. Insulin Resistance and Endothelial Dysfunction

Insulin resistance is a key mechanistic link between SO and atherosclerosis. As a primary target tissue for insulin action, muscle mass reduction directly decreases insulin sensitivity and exacerbates IR. The occurrence of SO is often accompanied by fat infiltration in skeletal muscles, with adipose tissue secreting lipotoxic factors that synergistically promote atherosclerosis [125]. Muscle tissue improves metabolic health through glucose uptake and lipid oxidation, but when muscle mass decreases (especially when it is infiltrated by fat), its metabolic buffering capacity is weakened [75]. A reduction in muscle mass can downregulate the expression of glucose transporter 4 (GLUT4), impairing insulin-mediated glucose uptake, which in turn induces hyperglycemia [126]. Persistent hyperglycemia accelerates the glycation of large molecules in the circulation, resulting in the formation of advanced glycation end products (AGEs). AGEs promote vascular endothelial oxidative stress, increase low-density lipoprotein cholesterol (LDL-C) oxidation, and worsen vascular stiffening and calcification [127]. IR further promotes lipolysis in adipose tissue, increasing free fatty acid (FFA) levels, leading to lipid metabolism disturbances and accelerating the progression of atherosclerosis [72,80,128]. IR also contributes to mitochondrial dysfunction and promotes muscle protein degradation by activating the ubiquitin-proteasome system (UPS), creating a vicious cycle [79].

Clinical and animal studies indicate that in the pathological process of SO, partial inactivation of the AMPK pathway not only enhances fatty acid-induced nuclear factor kappa B (NF-κB) activation and mammalian target of rapamycin (mTOR) signaling but also leads to the accumulation of lipid metabolites and ceramides. This, in turn, activates serine/threonine kinases, resulting in the phosphorylation of serine/threonine sites on insulin receptor substrates (IRSs), thereby impairing insulin signaling and promoting the development of IR [129,130]. IR weakens endothelial cell responses to insulin, decreases endothelial nitric oxide synthase (eNOS) activity, reduces nitric oxide (NO) synthesis, and inhibits vascular relaxation, thereby promoting the formation of atherosclerotic plaques [13,14]. Additionally, IR-related hyperinsulinemia can interfere with the IRS-1/PI3K/Akt pathway, leading to endothelial dysfunction [131,132]. Hyperinsulinemia also drives the conversion of vascular smooth muscle cells (VSMCs) from a contractile to a synthetic phenotype. In synthetic VSMCs, the activity of acid cholesterol ester hydrolase is reduced, causing intracellular accumulation of cholesterol and its esters and increasing the risk of muscle-derived foam cell formation, thereby increasing susceptibility to atherosclerosis [133,134].

### 7.3. Chronic Low-Grade Inflammation and Oxidative Stress

Chronic low-grade inflammation and oxidative stress are also key mechanisms linking SO with atherosclerosis [82]. A decrease in muscle mass, combined with obesity, promotes the release of inflammatory factors, leading to endothelial dysfunction [97]. SO patients often exhibit gut microbiota dysbiosis, which, through the bile acid-FXR signaling pathway, reduces the production of anti-inflammatory short-chain fatty acids (SCFAs), thereby exacerbating inflammation, accelerating muscle loss, and promoting atherosclerosis [135,136]. With aging and reduced physical activity, the process of adipocyte hypertrophy accelerates, driving macrophage infiltration and stimulating adipocytes and immune cells to secrete proinflammatory cytokines (such as TNF-α, IL-6, IL-1β, and IFN-γ) while inhibiting the expression of anti-inflammatory cytokines (such as IL-15) [137,138]. This imbalance between proinflammatory and anti-inflammatory factors can result in chronic low-grade inflammation, exacerbating vascular inflammation and oxidative stress levels [138]. Adipocytes and macrophages also secrete monocyte chemoattractant protein-1 (MCP-1) [139], which recruits monocytes to invade the vascular endothelium, where they differentiate into macrophages and engulf oxidized low-density lipoprotein (ox-LDL), forming foam cells and promoting plaque formation. Persistent high MCP-1 levels create an inflammatory microenvironment that destabilizes plaques and increases the risk of rupture [140,141]. Studies have confirmed that SO patients present a “high-inflammatory phenotype,” with elevated biomarkers of inflammation, such as white blood cell count (WBC), C-reactive protein (CRP), the neutrophil-to-lymphocyte ratio (NLR), and the urinary 8-iso-prostaglandin F2α (8-iso-PGF2α). Concurrently, studies have shown that the Aggregate Index of Systemic Inflammation (AISI) and the Systemic Inflammation Response Index (SIRI) exert significant modulatory effects on the relationship between SO and arterial stiffness, potentially serving as risk stratification tools [72]. The data collectively demonstrate that oxidative stress levels in SO patients are markedly elevated compared with those in healthy-weight individuals and are intricately linked to the onset and progression of atherosclerosis [76,81,142]. Moreover, SO causes mitochondrial impairment and enhances NADPH oxidase (NOX) activity, resulting in the overproduction of reactive oxygen species (ROS). Reactive oxygen species (ROS) facilitate atherosclerosis progression by triggering vascular smooth muscle cell (VSMC) death and excessive collagen accumulation, thereby diminishing arterial flexibility [76,81,142].

### 7.4. Dyslipidemia and Vascular Injury

Patients with SO commonly exhibit pronounced lipid metabolism disorders, a pathological state that not only disrupts systemic metabolic homeostasis but also directly accelerates the progression of atherosclerosis. In SO, excessive visceral fat accumulation is closely associated with characteristic dyslipidemia, such as hypertriglyceridemia and reduced high-density lipoprotein cholesterol (HDL-C), which jointly promote atherosclerosis development [16]. Kim et al. further demonstrated a positive correlation between the degree of skeletal muscle fat infiltration and decreased HDL-C, as well as increased low-density lipoprotein cholesterol (LDL-C), triglyceride (TG), and small dense LDL-C (sdLDL-C) levels [143]. Insulin resistance can also exacerbate atherogenic lipid profiles by inhibiting hepatic glycogen synthesis while enhancing hepatic lipogenesis [143].

Dyslipidemia contributes to vascular injury through multiple mechanisms, including promoting lipid deposition within the vessel wall, inducing vascular narrowing, and facilitating plaque formation [17]. It also drives ectopic lipid accumulation in the liver, skeletal muscle, and perivascular regions, triggering metabolic diseases such as diabetes and NAFLD while impairing vascular structure [127]. Moreover, elevated blood viscosity resulting from dyslipidemia further disrupts hemodynamics, aggravating vascular dysfunction. Therefore, improving lipid metabolism in SO patients may represent a potential intervention strategy to slow atherosclerosis progression and reduce cardiovascular risk.

### 7.5. Vicious Cycles of Sarcopenia, Obesity, and Atherosclerosis

**Figure 2:**
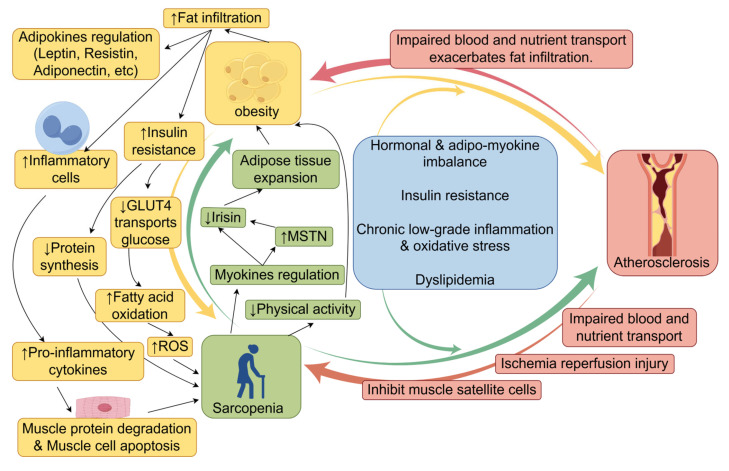
The vicious cycle of sarcopenia, obesity, and atherosclerosis (created with figdraw.com). Sarcopenia, obesity, and atherosclerosis involve multiple negative interactions, forming a self-reinforcing vicious cycle. Abbreviations: Adipo-myokines, adipokines, and myokines; GLUT4, glucose transporter type 4; ROS, reactive oxygen species; MSTN, myostatin.↑, Increased expression or enhanced effect; ↓, expression reduction or diminished effect; Other arrows, Pathogenic mechanism of the next step.

The aforementioned mechanisms indicate that SO contributes to atherosclerosis via various pathways, including hormonal/adipo-myokine imbalance, insulin resistance, chronic inflammation, and lipid metabolism disorders, collectively establishing a complex pathological network that facilitates the onset and progression of atherosclerosis. These mechanisms are interconnected, creating causal loops (e.g., insulin resistance exacerbates inflammation and lipid metabolism abnormalities, whereas inflammation further impairs insulin signaling pathways), collectively propelling the progression of vascular pathology from initial endothelial injury to advanced plaque formation. Furthermore, sarcopenia, obesity, and atherosclerosis exhibit multiple negative interactions, creating a self-reinforcing vicious cycle (Figure 2).

Sarcopenia and obesity are associated with multiple common risk factors, including age, inflammation, insulin resistance, and diminished physical activity, which mutually exacerbate one another [144,145,146]. The precise processes governing interactions between muscle and adipose tissue have not been fully elucidated, and several potential pathways have been proposed. Sarcopenia can clinically result in diminished physical activity and lowered caloric expenditure, thereby increasing the risk of obesity [19]. At the molecular level, myokines—cytokines released by muscle—interact with various organs, including adipose tissue, the liver, the brain, bone, and the immune system. Myokines not only facilitate muscle regeneration but also orchestrate energy metabolism by modulating glucose and fatty acid absorption and use [147]. With aging, the secretion levels of the majority of myokines diminish. Reduced irisin levels in elderly individuals are associated with both sarcopenia and age-related adipose tissue growth [148]. Myostatin (MSTN), a highly researched myokine, significantly influences both muscle and fat tissues [149]. MSTN expression is elevated in obese animal models and populations, presumably facilitating fat accumulation and muscle atrophy by suppressing irisin production, thereby increasing the risk of SO in the elderly [150].

Conversely, with increasing age, adipose tissue often deposits ectopically in nonadipose organs, including muscle, liver, and pancreas [151]. The accumulation of lipids and associated metabolites in muscle tissue is referred to as myosteatosis. These lipids can disrupt insulin signaling pathways, impairing GLUT4 transport function and thereby diminishing skeletal muscle glucose absorption [152]. Compensatory increases in fatty acid oxidation intensify mitochondrial overload, hinder electron transport chain functionality, disturb oxidative phosphorylation, increase reactive oxygen species (ROS) production, and ultimately result in muscular atrophy [48]. Impaired insulin signaling inhibits protein synthesis, directly leading to myofibrillar atrophy and hastening muscle degradation. Moreover, adipose tissue expansion results in the recruitment of substantial quantities of inflammatory cells (e.g., macrophages, inflammatory T cells, and mast cells) to adipose and muscle tissues. Macrophages transition from the anti-inflammatory M2 phenotype to the proinflammatory M1 phenotype, releasing proinflammatory mediators such as IL-6, TNF-α, IL-1β, MCP-1, CCR2, and CCR5 [153]. These substances promote muscle protein degradation and muscle cell apoptosis, hence intensifying muscle atrophy [153,154].

To present, over 600 adipokines have been identified. These cytokines, produced by adipocytes or the extracellular matrix, exert multifaceted regulatory effects on muscle tissue [155]. For instance, leptin promotes myoblast proliferation and increases myofibrillar cross-sectional area, thereby enhancing muscle regeneration [156,157]. Resistins, conversely, inhibit myogenesis and are particularly associated with age-related muscle strength decline in older men [158]. Adiponectin, an anti-inflammatory adipokine, improves glucose and lipid metabolism in aging muscles, enhances insulin sensitivity, and plays a crucial role in maintaining skeletal muscle structure and regeneration [155,158].

Atherosclerosis-associated vascular lesions hasten muscle atrophy and functional deterioration by obstructing blood flow and nutrient delivery while concurrently intensifying fatty infiltration [159,160,161,162]. A higher degree of ischemia correlates with a more significant reduction in muscle mass and function, along with increased muscle fatty infiltration [163]. A cohort study revealed that women with asymptomatic peripheral artery disease (ankle-brachial index < 0.90) presented reduced physical activity levels, diminished walking speeds, and compromised balance [164]. Studies indicate that individuals with peripheral artery disease (PAD) experience ischemic episodes in the calf muscles during walking, followed by reperfusion during periods of rest. The ischemia-reperfusion phenomenon is associated with significant generation of reactive oxygen species (ROS), including superoxide anions and hydrogen peroxide. This mechanism compromises muscle fiber integrity and mitochondrial functionality, increases oxidative stress, and triggers apoptosis [165]. Electron imaging of calf muscles in patients with peripheral artery disease has revealed that mitochondrial deformation is indicative of significant dysfunction [166]. Moreover, vascular endothelial dysfunction leads to diminished nitric oxide generation and increased inflammatory responses, which can hinder satellite cell proliferation and compromise muscle repair ability [167,168].

In summary, sarcopenia, obesity, and atherosclerosis theoretically form a complex vicious cycle. Compared with examining sarcopenia and obesity in isolation, delving into the association between SO and atherosclerosis and its underlying mechanisms holds greater practical importance for clinical practice.

## 8. Research Challenges and Future Directions

### 8.1. Current Research Challenges and Limitations

SO is a multifaceted clinical syndrome that transcends a mere amalgamation of obesity and sarcopenia. SO markedly hastens the initiation and progression of atherosclerosis via various mechanisms, including hormonal imbalances, deregulation of adipokines and myokines, insulin resistance, chronic low-grade inflammation, and abnormalities in lipid metabolism. The intricate, interdependent relationships between sarcopenia, obesity, and atherosclerosis are often overlooked and necessitate deeper clarification of the underlying mechanisms involved. The current study revealed correlations between SO and atherosclerosis across various aspects. The association between SO and coronary atherosclerosis is relatively consistent, but the connections with carotid atherosclerosis, arterial stiffness, and 10-year ASCVD risk exhibit variability. The variability is chiefly attributed to disparities in study methodology, diagnostic criteria, comorbidities (including type 2 diabetes, nonalcoholic fatty liver disease, and HIV infection), and sex.

Current research still faces multiple challenges. First, the diagnostic criteria remain inconsistent: different international organizations (e.g., ESPEN/EASO, JWGSO, and AOASO/IAGG-AOR) employ varying diagnostic indicators. Muscle mass assessment involves multiple parameters, such as ALM/W, SMM/W, ASM/BMI, and ASMI, whereas obesity assessment encompasses BMI, waist circumference, body fat percentage, and visceral fat area (VFA). This inconsistency hinders the comparability of research findings. Regions must validate applicable indicators tailored to their local populations. Currently, most studies employ single muscle or obesity metrics without integrating grip strength or physical function assessments, potentially overlooking at-risk groups such as those in the “possible sarcopenic obesity” stage [52]. While ASM/VFA has demonstrated strong associations with atherosclerosis in research, its clinical application remains constrained by the lack of standardized testing protocols and clear diagnostic thresholds.

Second, causality remains unestablished: existing studies predominantly employ cross-sectional designs with limited cohort research, preventing the confirmation of causal relationships between SO and atherosclerosis [76].

Additionally, the research scope is limited. There is insufficient focus on peripheral arterial atherosclerosis, such as in the lower limbs, renal arteries, and cerebral arteries. Moreover, the relative contributions of sarcopenia and obesity to atherosclerosis progression remain contentious, with some studies supporting the dominant role of sarcopenia and others emphasizing the key impact of obesity. Further, the majority of existing studies are performed on Asian populations; hence, the analysis and conclusions of this review may be constrained by the regional distribution of the study population.

Finally, there is a lack of population-specific studies. Some research suggests that sex differences (e.g., the association between SO and ASCVD risk is stronger in elderly women than in men) [34], which may be related to hormonal or body composition distribution differences. The modifying effects of comorbidities on the SO–Atherosclerosis relationship have not been fully elucidated. There is a lack of stratified analyses based on sex and comorbidities.

### 8.2. Future Research Directions

Based on current research progress, challenges, and limitations, future studies can be further developed in these areas.

For diagnostic criteria, countries or regions should establish sarcopenic obesity diagnostic standards tailored to local population characteristics or further validate the applicability of existing standards to their populations, thereby promoting diagnostic standardization. The clinical value of novel indicators such as ASM/VFA can be further validated, and efforts should be made to incorporate these metrics into consensus diagnostic standards.

Regarding research methodologies, attention should be directed to possible sarcopenic obesity. Further studies should examine the health implications of obesity combined with diminished grip strength or physical function, comparing these outcomes with individuals experiencing muscle mass decline to facilitate early intervention. Additionally, attention should be paid to atherosclerosis in peripheral arteries such as the lower limb arteries, renal arteries, and cerebral arteries, systematically comparing the differential effects of SO on atherosclerosis in different vascular locations. Simultaneously, stratified analyses by gender and comorbidities (e.g., type 2 diabetes, non-alcoholic fatty liver disease) should clarify how the association between SO and atherosclerosis manifests across different subgroups, thereby enabling personalized interventions. Prospective cohort studies should also be conducted to establish the causal relationship between SO and atherosclerosis.

In terms of mechanism research, the interactions and synergistic mechanisms among muscle mass/function decline, fat accumulation, and atherosclerosis should be explored in greater depth. This will provide new strategies for early warning of atherosclerosis and the prevention and treatment of SO–Atherosclerosis comorbidity.

## Data Availability

Not applicable.
